# Activation of human RNA lariat debranching enzyme Dbr1 by binding protein TTDN1 occurs though an intrinsically disordered C-terminal domain

**DOI:** 10.1016/j.jbc.2023.105100

**Published:** 2023-07-26

**Authors:** Nathaniel E. Clark, Adam Katolik, Pascal Gallant, Anastasia Welch, Eileen Murphy, Luke Buerer, Christoph Schorl, Nandita Naik, Mandar T. Naik, Stephen P. Holloway, Kristin Cano, Susan T. Weintraub, Katherine M. Howard, P. John Hart, Gerwald Jogl, Masad J. Damha, William G. Fairbrother

**Affiliations:** 1 Department of Molecular Biology, Cell Biology, and Biochemistry, Brown University, Providence, Rhode Island, USA; 2 Department of Chemistry, McGill University, Montreal, Quebec, Canada; 3 Department of Biochemistry and Structural Biology, University of Texas Health Science Center, San Antonio, Texas, USA; 4 Department of Biomedical Sciences, School of Dental Medicine, University of Nevada-Las Vegas, Las Vegas, Nevada, USA

**Keywords:** RNA, lariats, introns, debranching enzyme, metalloenzymes, metallophosphodiesterase, phosphorothioate, branched RNA, crystal structure

## Abstract

In eukaryotic cells, the introns are excised from pre-mRNA by the spliceosome. These introns typically have a lariat configuration due to the 2′-5′ phosphodiester bond between an internal branched residue and the 5′ terminus of the RNA. The only enzyme known to selectively hydrolyze the 2′-5′ linkage of these lariats is the RNA lariat debranching enzyme Dbr1. In humans, Dbr1 is involved in processes such as class-switch recombination of immunoglobulin genes, and its dysfunction is implicated in viral encephalitis, HIV, ALS, and cancer. However, mechanistic details of precisely how Dbr1 affects these processes are missing. Here we show that human Dbr1 contains a disordered C-terminal domain through sequence analysis and nuclear magnetic resonance. This domain stabilizes Dbr1 *in vitro* by reducing aggregation but is dispensable for debranching activity. We establish that Dbr1 requires Fe^2+^ for efficient catalysis and demonstrate that the noncatalytic protein Drn1 and the uncharacterized protein trichothiodystrophy nonphotosensitive 1 directly bind to Dbr1. We demonstrate addition of trichothiodystrophy nonphotosensitive 1 to *in vitro* debranching reactions increases the catalytic efficiency of human Dbr1 19-fold but has no effect on the activity of Dbr1 from the amoeba *Entamoeba histolytica*, which lacks a disordered C-terminal domain. Finally, we systematically examine how the identity of the branchpoint nucleotide affects debranching rates. These findings describe new aspects of Dbr1 function in humans and further clarify how Dbr1 contributes to human health and disease.

The RNA lariat debranching enzyme, Dbr1, hydrolyzes the 2′-5′ bond in intron lariats produced by the spliceosome (1, 2). These intron lariats are rapidly degraded by exonucleases following Dbr1 hydrolysis. The *DBR1* gene was first identified in yeast genetic screens aimed at identifying factors which restrict Ty1 retrotransposition (3). *Saccharomyces cerevisiae* develop a modest growth defect with Dbr1 ablation, and *Saccharomyces pombe* exhibit a more severe growth defect attributed to the greater number of intron-containing genes in the latter strain (4).

In metazoa, *DBR1* is an essential gene, and Dbr1 knock-out mice are inviable (5, 6). Loss of Dbr1 impairs class-switch recombination of immunoglobulin genes, promotes oncogenesis, impairs HIV replication, and compromises cell-intrinsic immunity to viruses (5, 7, 8, 9, 10, 11). Inhibition of Dbr1 could be protective from amyotrophic lateral sclerosis and other neurodegenerative diseases linked to toxic aggregates of TDP-43. Excess lariats can reduce the toxicity of TDP-43 aggregates (12, 13, 14).

Dbr1 is a metallophosphoesterase (MPE) enzyme. MPEs are nucleases and phosphatases that use a two-metal ion active site cluster formed by a highly conserved GNHD/E motif. In Dbr1, this motif contains an Asn(N) which coordinates an Fe^2+^ ion, a catalytic His (H) which protonates the 2′ leaving group, and a structural Asp (D) that forms solvent-inaccessible hydrogen bonds with a neighboring beta-strand. Dbr1 enzymes are unique MPEs because they have a Cys (C) residue in place of an Asp (D) that is conserved in every other MPE. This substitution leads to a stable Zn^2+^-Cys bond that has been observed in crystal structures from two independent groups. This bond protects the adjacent Fe^2+^ co-factor from oxidation (15, 16, 17).

Metazoan Dbr1 homologs (*i.e.*, human, mouse) contain ∼200 aa at the C terminus that are absent in yeast or amoeba Dbr1 enzymes. A nuclear localization sequence is located in this region (residues 511–528) (18, 19), but no other biological functions are ascribed to this region, and no structural data are available for human Dbr1. Crystal structures are available for *Entamoeba histolytica* Dbr1, and in these structures, the C terminus is ∼50 aa and plays a structural role only, consisting of three alpha helices ∼8 to 20 aa which lay along the backside of the enzyme, distal to the active site and RNA binding surfaces (16, 20). All disease-causing human Dbr1 mutations identified to date affect buried residues in the N-terminal MPE domain and are predicted to destabilize the catalytic domain (21).

Dbr1 forms a complex with the homologous, noncatalytic protein Drn1 in yeast and humans. In yeast, this interaction enhances the turnover of branched RNA (22). In human cells, Dbr1 immunoprecipitates with the human Drn1 homolog (Cwf19-L1) (18). Mutations in Drn1 homolog are linked to cerebellar ataxia (23). Additionally, several high-throughput co-IP-MS screens have identified interactions between human Dbr1 and the protein trichothiodystrophy nonphotosensitive 1 (TTDN1) (24, 25). TTDN1 is a protein previously implicated in trichothiodystrophy (26) but has no known biochemical function. The effects of Drn1 or TTDN1 interactions with Dbr1 on either activity or substrate specificity are unknown.

Here, we examine biochemical and biophysical properties of human Dbr1. We examine how the C-terminal domain contributes to Dbr1 activity and stability. We determine the metal cofactors required for human Dbr1 catalysis. We demonstrate that TTDN1 protein levels are determined by Dbr1 levels in human cells and that Drn1 and TTDN1 interact directly with Dbr1. We find that TTDN1 increases the catalytic efficiency of Dbr1 by 19-fold but does not alter the substrate specificity of Dbr1. These data clarify the requirements for lariat metabolism in humans and offer new mechanistic details on the role of Dbr1 in human health and disease.

## Results

### The C-terminus of human Dbr1 is intrinsically disordered

**Figure 1:**
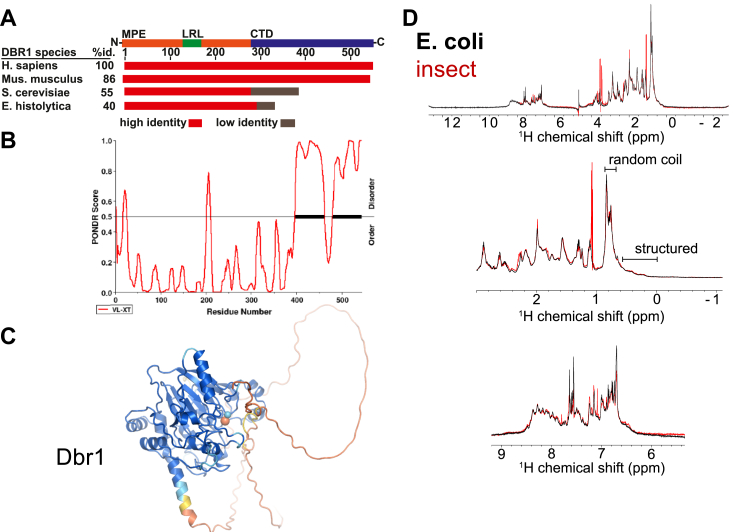
**Human Dbr1 has a disordered C-terminal domain.***A*, summary of primary sequence alignment of Dbr1 homologs from human, mice, yeast, and amoeba. The catalytic metallophosphoesterase (MPE) domain is conserved across phyla. The lariat-recognition loop (LRL) is unique to Dbr1 enzymes. Metazoan Dbr1 enzymes have a long, conserved C-terminal domain that is absent in lower eukaryotes. *B*, the C-terminal domain is predicted to be disordered based on the primary sequence (PONDR analysis). *C*, predicted 3 dimensional structure of human Dbr1 colored by pLDDT score, with *blue* = high confidence and *red* = low confidence. The active site metals are shown as *spheres*. The C terminus is *red* and lacks secondary structure. *D*, 1D proton NMR confirms that human Dbr1 contains random-coil polypeptide when expressed in either *E. coli* (*black spectra*) or Sf9 insect cells (*red spectra*). The methyl region is shown (*middle*) with the regions corresponding to random coil and structured polypeptide labeled. The amide region is identical for both preparations (*bottom*).

Analysis of the human Dbr1 amino acid sequence with BLAST (27, 28) reveals a MPE domain spans approximately the first 300 aa residues, and aligns to many other MPE enzymes, while the C-terminal domain (residues ∼350–544) has no homology to any annotated functional domain (Fig. 1*A*). The order/disorder predictor PONDR uses the relative frequency of charged and hydrophobic residues to forecast the presence of disordered regions (29), and PONDR clearly predicted that the C-terminal ∼150 aa of Dbr1 are unstructured (Fig. 1*B*). Consistent with this PONDR prediction, unstructured polypeptide is present in the C-terminal domain of an hDbr1 Alpha-Fold model (Fig. 1*C*) (30, 31).

We tested this prediction by expressing and purifying Dbr1 from a prokaryotic (*Escherichia* *coli*) and a eukaryotic (*Sf9* insect cell) expression system, and we performed 1D-proton nuclear magnetic resonance (NMR) on the purified protein samples (Fig. 1*D*, top). Both samples gave intense peaks from methyl groups (0.8–0.5 PPM), consistent with the presence of random coil polypeptide (Fig. 1*D*, middle) (32). The amide and aliphatic proton regions were indistinguishable for the two samples. We concluded that hDbr1 had the same folded state when expressed in prokaryotic or eukaryotic cells and that the presence of the random coil was not an artifact of heterologous expression in prokaryotic cells. A circular dichroism spectra of *E. coli* expressed hDbr1 is presented in Fig. S1*B*.

### The disordered C terminus stabilizes Dbr1 but is not required for activity

**Figure 2:**
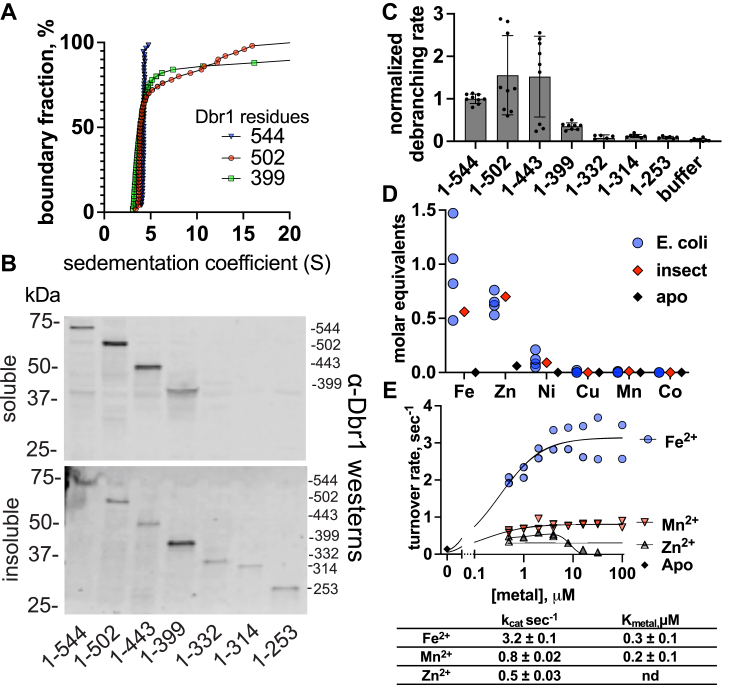
**Role of the Dbr1 C-terminal domain.***A*, analytical ultracentrifugation shows that Dbr1 proteins with C-terminal truncations aggregate. Full-length Dbr1 (aa. 1–544) is monodisperse, while Dbr1-502 and DBr1-399 self-associate to produce species with very large S-values. *B*, Western blot of soluble and insoluble *E. coli* lysates shows soluble expression residues 1 to 399 and longer (*top blot*, soluble lysate), and insoluble expression for residues 1 to 332 and shorter (*bottom blot*, insoluble lysate). *C*, debranching activity is measured for residues 1 to 399 and longer variants only. *D*, purified Dbr1 proteins contain ∼1 Fe and ∼0.7 Zn ions per monomer following expression in *E. coli* or Sf9-insect cells. Metal-free Apo-Dbr1 has 0.06 Zn ions per monomer and <0.00 equivalents of Fe, Ni, Cu, Mn, or Co. *E*, anaerobic metal reconstitution assays with apo hDbr1. Maximal rates were 3.2 ± 0.1 s^−1^ with Fe^2+^, 0.8 ± 0.02 s^−1^ with Mn^2+^, and 0.5 ± 0.03 s^−1^ with Zn^2+^.

To measure how the disordered C terminus contributed to Dbr1 activity or stability, we created a series of C-terminal truncations by introducing stop-codons after residues 253, 314, 332, 399, 443, and 502. For full-length (1–544) Dbr1, Dbr1-502, and Dbr1-399, we analyzed purified proteins with analytical ultracentrifugation. Full-length Dbr1 was monodisperse, with a narrow distribution of S-values centered around 4.3 (Fig. 2*A*). Dbr1-502 and Dbr1-399 showed considerable aggregation, with ∼20% of the samples sedimenting at very large S-values.

To test if the C-terminal domain was required for debranching activity, we expressed full-length and truncated Dbr1 proteins in *E. coli*. We measured the protein levels of Dbr1 in the soluble and insoluble lysate fractions with a Western blot and performed debranching assays on the soluble fractions (Fig. 2, *B* and *C*). Residues 1 to 399 were sufficient for both soluble expression and activity, and truncations with fewer than 399 residues were neither soluble or active (Fig. 2, *B* and *C*). Some quantity of full length-, 502-, and 433-Dbr1 was present in the insoluble fraction, which could represent incomplete lysis/extraction, or misfolded protein produced during the induction phase of the overexpression experiments. However, these samples displayed no signs of aggregation or precipitation after extraction. The entire experiment was repeated three times, and the rates were normalized to the debranching rate of full-length Dbr1 (Fig. 1*C*). Due to variability in the expression levels of the individual fragments, we were unable to decouple possible changes in specific activity from changes in protein levels in the lysates.

### Metal-dependence of human Dbr1

To understand the metal cofactor requirements for human Dbr1 catalysis, we first examined which metals were present in Dbr1 samples after purification from *E. coli* or Sf9 insect cells using inductively coupled plasma mass-spectrometry. In four samples of *E. coli*-expressed Dbr1 and one sample of Sf9-expressed Dbr1 (Fig. 2*D*), Fe and Zn were the most abundant metals, with a mean of 1 Fe and 0.7 Zn ions per Dbr1 monomer. A small quantity of Ni was detected (∼0.1 Ni ion per monomer) which we attribute to the C-terminal His-tag. No exogenous metal ions were added to growth media, lysis, or purification buffers.

To systematically examine the ability of Fe, Zn, and Mn to support Dbr1-mediated debranching, we prepared metal-free apo Dbr1 by exposing the purified enzyme to high concentrations of metal chelators. Apo Dbr1 was devoid of activity as expected (Fig. 2*E*, metal = 0 point) and contained 0.06 Zn ions per monomer and <0.00 Fe, Mn, Ni, Cu, or Co ions per monomer (Fig. 2*D*, black diamonds). We reconstituted apo-Dbr1 with Fe^2+^, Mn^2+^, and Zn^2+^ under anaerobic conditions to prevent oxidation of Fe^2+^ to Fe^3+^. After a 10-min incubation, the mixtures of metals+apo-Dbr1 were mixed with a dark-to-bright fluorescent bRNA reporter substrate (33), and steady-state reactions velocities were measured in real time (Fig. 2*E*). Fe^2+^ reconstituted apo-Dbr1 had the highest debranching rate, 3.2 s^−1^. Mn^2+^ rates were 0.8 s^−1^, and Zn^2+^ rates were 0.5 s^−1^. Unlike Fe^2+^ and Mn^2+^, Zn^2+^ rates decreased above 8 μM Zn^2+^.

### Dbr1 is the key determinant of TTDN1 protein levels

Realizing that the C-terminal tail was not required for debranching, we hypothesized that it may participate in important protein:protein interactions. To determine if loss of Dbr1 disrupted other proteins, networks, or pathways, we generated two Dbr1-KO cell lines from HEK 293 cells. To control artifacts of cell line generation, we 'rescued' Dbr1 KO cells with Dbr1 transfection. To control for transfection artifacts, we performed separate transfections with red fluorescent protein (RFP) expression plasmids.

**Figure 3:**
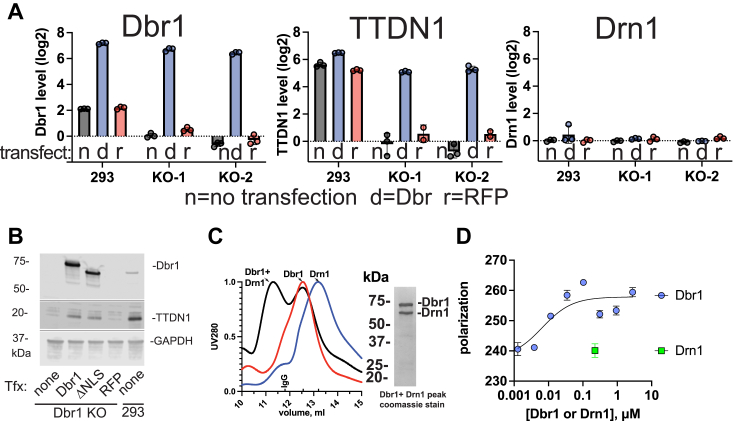
**Dbr1 interacts with TTDN1 and Drn1.***A*, whole-cell proteomics HEK293 cells with Dbr1 KO or Dbr1 overexpression reveal that TTDN1 protein levels are determined Dbr1 protein levels. The transfection conditions are indicated below the columns. *B*, Western blot validation of the proteomic results. The blotting antibodies are indicated on the right. ΔNLS refers to Dbr1 with the nuclear localization sequence deleted. The parent 293 cell line is in the rightmost lane. *C*, size-exclusion chromatography demonstrates that Dbr1 and Drn1 form robust complex. The elution volume of an IgG standard is indicated above the x-axis (∼150 kDa). A Coomassie-stained gel of the peak fraction is presented. *D*, the polarization of fluorescently labeled TTDN1 increases with increasing Dbr1 concentration, suggesting a direct interaction and a K_d_ of approximately 3 nM. No increase in polarization was observed when Drn1 was added to TTDN1. TTDN1, trichothiodystrophy nonphotosensitive 1.

We measured the whole-cell proteome of the 9-cell line/transfection combinations in triplicate with data-independent acquisition mass-spectrometry techniques. Endogenous Dbr1 was detected in 293 cells, but not in the Dbr1-KO cells. Dbr1-transfection raised Dbr1 levels, while RFP transfection did not (Fig. 3*A*, left and Fig. 3*B*). We searched the set of ∼5000 proteins with quantitative expression data for proteins that followed the expression pattern of Dbr1. The levels of a single protein, TTDN1, showed perfect correlation with Dbr1 levels. TTDN1 disappeared in Dbr1-KO cells and reappeared after Dbr1 transfection (Fig. 3*A*, middle). The TTDN1 mRNA levels were unchanged, with log2 fold change = −0.17, *p*_adj_ = 0.44. In contrast, the protein levels of Drn1, which co-IPs with Dbr1 in human cells, were unaffected by Dbr1 protein levels (Fig. 3*A*, right). Western blots validated these results (Fig. 3*B*). Thus, TTDN1 is unstable in the absence of Dbr1, which is not the case for Drn1.

### Interaction of Dbr1 with TTDN1 and Drn1

To examine the ability of Dbr1 to form protein:protein complexes with TTDN1 and Drn1, we expressed and purified these proteins from *E. coli*. 1D-proton NMR of TTDN1 indicated that TTDN1 was mostly disordered but contained some helical content (Fig. S1). We observed a drop in the amide region (8.2 ppm) at acidic pH (pH 4.5 *versus* 7.4), suggesting a collapse of structure at low pH. Circular dichroism of TTDN1 (pH 7.4) verified this helical content, with a minima around 220 nm (Fig. S1*B*). A predicted structure of TTDN1 also has short helical regions (Fig. S1*C*) (30, 31). A mixture of Dbr1 and Drn1 was fractionated on a size-exclusion column (Fig. 3*C*), a new peak appeared at larger apparent molecular weight, with a stoichiometry of 1:1 Dbr1 to Drn1 by denaturing gel electrophoresis (Fig. 3*C*). This complex peak elutes slightly before an ∼150 kDa IgG standard (indicated on X-axis, Fig. 3*C*), and at a volume distinct from free Dbr1 or Drn1.

We attempted to isolate a TTDN1:Dbr1 complex with size-exclusion chromatography. However, in repeated attempts, only a few percent of the TTDN1 protein loaded on the column could be identified in the postcolumn fractions. We surmised that TTDN1 bound nonspecifically to the column matrix, and we shifted towards a solution-based fluorescence polarization assay to measure TTDN1:Dbr1 binding. We titrated unlabeled Dbr1 into a fixed concentration (2.5 nM) of fluorescently labeled TTDN1 and observed a saturating dose–response curve, with a K_d_ of approximately 3 nM. Drn1 alone caused no increase in the polarization of labeled TTDN1 (Fig. 3*D*, *green square*).

### TTDN1 increases the catalytic efficiency of Dbr1

**Figure 4:**
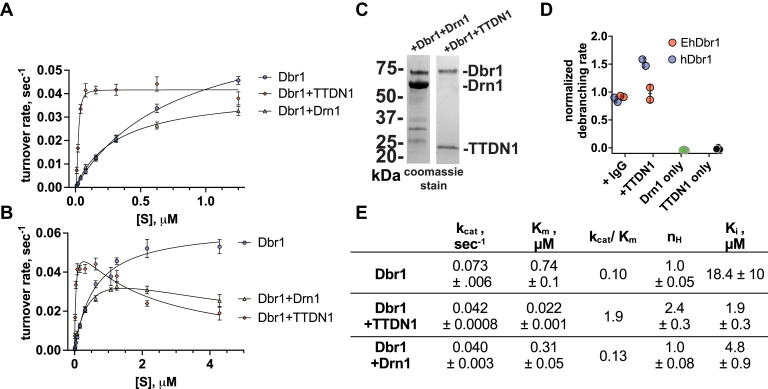
**Kinetic analysis of mixtures of Dbr1, Dbr1+Drn1, and Dbr1+TTDN1 assayed with a fluorescent branched RNA substrate.***A*, TTDN1 increases the affinity of Dbr1 for bRNA substrate. *B*, at higher substrate concentrations, the presence of Drn1 or TTDN1 cause substrate inhibition. *C*, Coomassie stained gel of the enzyme mixtures used in *A* and *B*. *D*, TTDN1 does not stimulate EhDbr1, which lacks a disordered C-terminal domain. IgG served as a control. Drn1 and TTDN1 have no debranching activity in the absence of Dbr1. *E*, results of curve fitting. The data in *A* were fit with a version of the Michaelis–Menten equation which includes a Hill-coefficient (n_H_), and in *B*, the equation was modified to include a substrate-inhibition term (K_i_). TTDN1, trichothiodystrophy nonphotosensitive 1.

We next examined how these protein:protein interactions affect the kinetic properties of Dbr1. We prepared mixtures of TTDN1:Dbr1 and Drn1:Dbr1 at 10:1 M ratio. These mixtures were assayed for debranching at different concentrations of fluorescent-branched RNA substrate (33) to determine K_m_ and k_cat_. The presence of TTDN1 increased the affinity for bRNA substrate, lowering the K_m_ by 33-fold, and resulting in maximal reaction velocities at only 20 nM substrate concentration. In contrast, Dbr1 or Drn1:Dbr1 mixtures required 1 to 2 μM substrate for maximal velocities. TTDN1 increased the catalytic efficiency of Dbr1 by 19-fold relative to Dbr1 alone (Fig. 4*E*). Drn1 did not enhance the kinetics of Dbr1 (Fig. 4, *A*, *B* and *E*). At higher substrate concentrations, TTDN1:Dbr1 and Drn1:Dbr1 mixtures displayed substrate inhibition (Fig. 4*B*) with K_i_-substrate values of 1.9 and 4.8 μM respectively (Fig. 4*E*). This was not seen for Dbr1 alone. A Coomassie stained denaturing gel of the mixtures is presented (Fig. 4*C*).

To determine if TTDN1 was stimulatory to a nonhuman Dbr1 ortholog which lacks a disordered C-terminal domain, we mixed TTDN1 with *E. histolytica* Dbr1 (EhDbr1). Purified IgG was used a control. We observed that only human Dbr1 was stimulated by TTDN1 (Fig. 4*D*), and IgG had no effect. We verified that Drn1 and TTDN1 have no debranching activity in the absence of Dbr1 (Fig. 4*D*).

### Branchpoint specificity of Dbr1 and TTDN1:Dbr1

**Figure 5:**
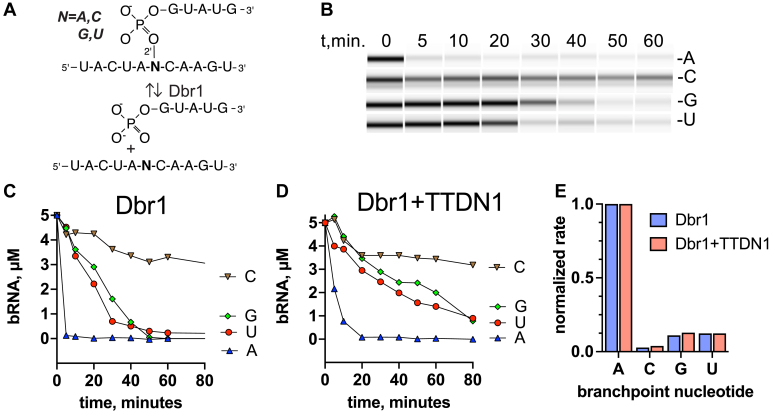
**Branchpoint specificity assay**. *A*, branched RNAs with A-, C-, G-, and U-branchpoint nucleotides were synthesized using solid-phase synthesis. *B*, cleavage of branched RNAs was measured with microcapillary electrophoresis. A pseudo-gel image is displayed for the Dbr1 cleavage reactions. *C*, quantification of Dbr1 cleavage data. *D*, quantification of Dbr1+TTDN1 cleavage data. *E*, the rates of bRNA cleavage were estimated from the slopes in *C* and *D* and normalized to the rates for *A*-branchpoint RNA. TTDN1, trichothiodystrophy nonphotosensitive 1.

To systematically examine the ability of Dbr1 to cleave branched RNA with different branchpoint nucleotides, we synthesized 16-mer branched RNAs with A, C, G, and U branchpoint substitutions (Fig. 5*A*). We measured the time course of 16-mer branched RNA hydrolysis with microcapillary electrophoresis (Fig. 5*B*). We found that A-branchpoints were cleaved rapidly with 100% cleavage at the first timepoint. Observed rates (k_obs_, h^−1^) were estimated from the plots of product *versus* time (Fig. 5, *C* and *D*). The rates were 12 h^−1^ (A), 0.33 h^−1^ (C), 1.3 h^−1^ (G), and 1.4 h^−1^ (U) for Dbr1, and 7 h^−1^ (A), 0.26 h^−1^ (C), 0.88 h^−1^ (G), and 0.85 h^−1^ (U) for TTDN1:Dbr1. Thus, G- and U-branchpoints were cleaved at ∼10% the rate of A-branchpoints, and C-branchpoints were cleaved at ∼3% the rate of A-branchpoints (Fig. 5*B*). Normalization of the k_obs_ to that of A-branchpoints reveals that TTDN1 does not affect branchpoint specificity (Fig. 5*E*). Based on EhDbr1 structures, the C-branchpoint is predicted to have poorer base stacking and steric clashes relative to the other branchpoint nucleotides (20).

## Discussion

Although Dbr1 plays a fundamental role in gene expression and mRNA splicing by hydrolyzing the lariat bonds produced by the spliceosome, mechanistic details as to precisely how loss of Dbr1 impairs viral immunity, oncogenesis, or viral replication are lacking. Because Dbr1 active site residues are conserved from *E. histolytica* (EhDbr1) to *S. cerevisiae* (yDbr1), to human, homology models of human Dbr1 based on the *E. histolytica* crystal structures (16, 17, 20) help explain structure-function relationships in human Dbr1 (21).

Human Dbr1 is a challenging target for structural studies. In parallel with the experiments presented in this manuscript, we performed extensive crystal screening of full-length, truncated, and proteolyzed human Dbr1. We failed to identify a single crystallization condition. We attribute this failure to the presence of the disordered C-terminal domain (Fig. 1). While this domain is dispensable for Dbr1 activity (Fig. 2*B*), purified Dbr1 proteins with C-terminal truncations self-associated and were of limited use in structural studies (Fig. 2*A*). The mass of Dbr1 (63 kDa) presents challenges for NMR-based structural determination. A 'divide and conquer' approach, where smaller pieces of Dbr1 are analyzed individually with NMR was hindered by the poor hydrodynamic properties of truncated Dbr1 proteins (Fig. 2*A*).

Previous work showed that highly active EhDbr1 and yDbr1 proteins co-purify with Fe^2+^/Zn^2+^ active site clusters following heterologous expression in *E. coli* (15, 16). These metal ions remain bound throughout cell lysis, two-column chromatographic purification, and overnight dialysis. Here we show that human Dbr1 also contains an Fe^2+^/Zn^2+^ active site cluster following purification from either metazoan or prokaryotic cell lines (Fig. 2*D*). Mixing Fe^2+^ with metal-free human Dbr1 resulted in maximal debranching rates. We previously observed that for EhDbr1, Zn^2+^ addition did not enhance debranching rates when combined with Fe^2+^ in anaerobic conditions (16). Mixtures of Fe^2+^ and Zn^2+^ restored full activity to apoenzymes (relative to that of the isolated holoenzyme), and these reconstituted species maintained activity in an aerobic environment, while the Fe^2+^-reconstituted Dbr1 lost all activity in an aerobic environment. These mixed metal reconstitutions were very challenging due to the different affinities of Fe^2+^ and Zn^2+^. The Fe^2+^ reconstitution rates were faster than the purified holoenzyme, and addition of Zn^2+^ only reduced the observed rates (shifting the Fe^2+^ curve downwards to the Zn^2+^ curve in Fig. 2*E*). We hypothesize that *in vivo*, Dbr1 enzymes use Fe^2+^, alone, or in combination with Zn^2+^. The stable Cys-Zn bond in the Dbr1 active site protects the beta-site Fe^2+^ from oxidation, and highly active Dbr1 with one Fe^2+^ and one Zn^2+^ per monomer can be purified under normal aerobic conditions (Fig. 4, *A* and *B*) (15, 16, 17).

Proteomic analysis of Dbr1 KO cells revealed a perfect correlation between TTDN1 and Dbr1 protein levels. TTDN1 was undetectable in Dbr1-KO cells, and TTDN1 re-appeared in Dbr1 add-back experiments (Fig. 3, *A* and *B*). We hypothesize that in the absence of Dbr1, TTDN1 is unstable and is rapidly degraded following translation (Fig. 3*B*). The presence of Dbr1 is sufficient to stabilize TTDN1 in human cell lines. The lack of a significant change in TTDN1 expression in Dbr1 KO cells (log2 fold change = −0.17. *p*_adj_ = 0.44) is consistent with a protein-stability phenomena. Both TTDN1 and Drn1 bind to Dbr1 in immunoprecipitation experiments (22, 24, 25, 34). We show that Dbr1 and Drn1 form a robust 1:1 complex which can be isolated with size-exclusion chromatography (Fig. 3*C*). Our attempts to isolate a TTDN1:Dbr1 complex with size-exclusion chromatography were complicated by non-specific binding of TTDN1 to the column matrix, so instead, we used fluorescence polarization to show that TTDN1 binds directly to Dbr1 *in vitro* (Fig. 3*D*).

**Figure 6:**
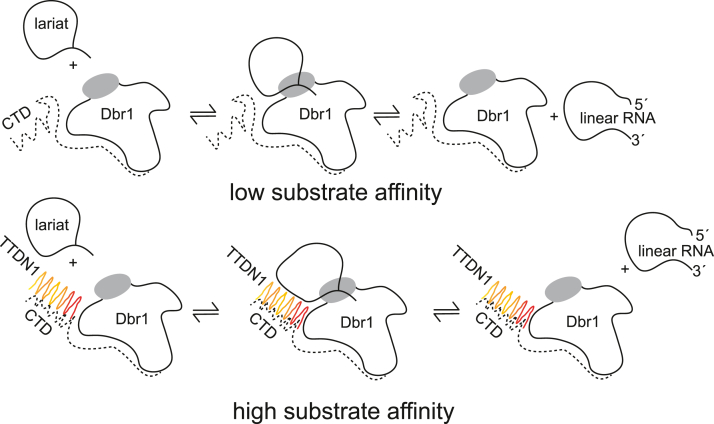
**TTDN1 increases the affinity of Dbr1 for bRNA when compared to Dbr1 only**. TTDN1 could bind to the Dbr1 C-terminal tail and help recruit RNA to the active site, which could also help remove product and therefore reduce product inhibition. Although Drn1 and Dbr1 form a robust complex, we did not observe an enhancement of debranching kinetics. TTDN1, trichothiodystrophy nonphotosensitive 1.

The addition of TTDN1 to Dbr1 debranching reactions resulted in a dramatic 19-fold increase in the catalytic efficiency of Dbr1, driven by a 33-fold reduction in K_m_. In contrast, Drn1 did not improve the catalytic properties of Dbr1 in our assays (Fig. 4, *A* and *E*). Both TTDN1 and Drn1 reactions demonstrated a high degree of substrate inhibition that was not observed in Dbr1-only reactions (Fig. 4*B*). We are unsure if this substrate inhibition should be considered an *in vitro* artifact of the 10:1 M excess of TTDN1/Drn1 to Dbr1 or to the relatively high concentrations of synthetic substrate used in the *in vitro* debranching assay (∼5 μM). Future experiments could examine lower ratios of TTDN1:Dbr1 and Dbr1:Dbr1 at high substrate concentrations to gain more insight into this substrate inhibition phenomena. Although we do not have an estimate of the lariat concentration in cells, cellular lariat concentrations of ∼10 to 100 nM would be sufficient to for Dbr1 to reach maximal debranching rates (Fig. 4*A*) given the observed K_m_ of 22 nM. We hypothesize that TTDN1 increases the catalytic efficiency of Dbr1 through binding to the disordered Dbr1 C-terminal domain. This is consistent with our result that EhDbr1 was not stimulated by TTDN1 addition. This suggests that TTDN1 stimulation is not due to a TTDN1:RNA substrate interaction but an interaction with hDbr1 that does not occur in EhDbr1. We hypothesize that TTDN1 interacts with the C-terminal tail (Fig. 6), noting that EhDbr1 and hDbr1 are 40% identical in the catalytic domain, but have no identity in the C-terminal domain which is absent in EhDbr1. However, this result could also be due to an interaction of TTDN1 with the catalytic domain.

TTDN1 binding could (i) help to recruit substrates to the Dbr1 activity site (consistent with the lower K_m_, Fig. 4*E*), (ii) prevent auto-inhibition from the C-terminal tail (consistent with trend towards increased activity of C-terminal truncations, Fig. 2*C*), or (iii) facilitate product removal from the active site, consistent with a recent crystal structure of EhDbr1 inhibited by a reaction product (35). Without further experiments or structural data, we cannot differentiate between these possibilities. Figure 6 presents a hypothetical model of how TTDN1 binding to Dbr1 could shift Dbr1 from a low to high substrate affinity state, consistent with the findings we report here.

Our systematic analysis of the relationship between branchpoint identity and Dbr1 hydrolysis demonstrates that A-branchpoints are the preferred substrates. G- and U-branchpoints are hydrolyzed at ∼10% the rate of A and C-branchpoints at ∼3%. This is similar to findings with yeast Dbr1 using ribozyme-generated bRNA (36). Another study compared the hydrolysis of A and C synthetic bRNAs and found that C were hydrolyzed more slowly (37). Here, we extend these findings to the human enzyme and quantify the kinetic rates through the use of defined concentrations of substrate and enzyme and multiple timepoints (Fig. 5*E*). In agreement with these results, C-branchpoints are enriched in stable intron lariats in *xenopus* oocytes (38).

In conclusion, hDbr1 contains significant quantities of unstructured random-coil polypeptide in an intrinsically disordered C-terminal domain. We demonstrate that Dbr1 binding partners such as TTDN1 modulate the kinetic properties of Dbr1 *in vitro*. This finding provides a basis to interpret biological data in which TTDN1 is present or absent, as we predict that loss of TTDN1 would impair lariat metabolism in cells or animal models. This raises the possibility that other factors could modulate lariat metabolism. Uncovering the networks of Dbr1 proteins that interact with Dbr1 might explain how loss of Dbr1 affects biological processes and diseases in humans, and these findings can serve as the basis for new testable hypotheses as to the role of TTDN1 and Drn1 in lariat metabolism.

## Experimental procedures

### Cloning, protein expression, and purification

#### Molecular biology

A codon-optimized human Dbr1 expression plasmid was synthesized by DNA2.0. Truncated hDbr1 expression constructs (1–502, 1–443, 1–399, 1–332, 1–314, 1–253, Δ511–528) were generated with PCR-based approaches (Phusion mutagenesis kit, Thermo). The Drn1 cDNA was purchased from Genecopoeia (Product ID: V1400; Cat #GC-OG08504) and subcloned into a modified pET vector along with a C-terminal 8x-HIS tag. A codon-optimized TTDN1 expression plasmids containing a C-terminal 8x-HIS tag was ordered from GenScript. All *E. coli* expression plasmids contained T7 promoters. For insect cell expression, the native human Dbr1 cDNA sequence was subcloned into the pFastBac vector along with a C-terminal 8x-HIS tag (Invitrogen Bac2Bac system).

#### Bacterial expression

T7-based expression plasmids were transformed into BL21 cells and grown in TB media (RPI) at 37 °C to ∼1 *A*_600_, at which point the temperature was reduced to 18 °C, and 1 mM IPTG was added to induce expression. After 18 h, the cells were harvested. 6 to 12 l of culture was produced for each purification.

#### Insect expression

Standard methods were used to obtain high-titer viral stocks (Invitrogen Bac2Bac system). Sf9 cells were cultured in EF 921 media (Expression Systems) at 27 °C in shake flasks. Cells at 2e6 cells/ml were infected with high-titer virus, a multiplicity of infection of 1. Forty-eight hours postinfection, the cells were harvested with centrifugation.

#### Dbr1 and Drn1 purification

For Dbr1, cells were resuspended in Ni-A buffer (=50 mM Tris pH 8, 250 mM NaCl, 1 mM Tris, 40 mM imidazole). For Drn1, cells were resuspended in BugBuster reagent (Novogene). Resuspended cells were sonicated, centrifuged to remove insoluble material, and applied to a 5 ml Hi-trap Ni Column (Cytiva). Proteins were eluted with a shallow gradient of Ni-B (=Ni-A+400 mM imidazole). Dbr1 fractions were diluted 10× in Q-A buffer (=20 mM Tris pH 8 50 mM NaCl 1 mM TCEP), loaded onto a Source 15 Q column, and eluted with a shallow gradient of Q-B buffer (=Q-A + 1 M NaCl). Drn1 Ni-fractions were concentrated and applied to a Superdex-200 sizing column (Cytiva) using SEC buffer (=50 mM Tris pH 8 100 mM NaCl 1 mM TCEP).

#### TTDN1 purification

TTDN1 was purified from inclusion bodies using the BugBuster reagent. Washed inclusion bodies were suspended in pH 11 CAPS (=100 mM CAPS pH 11, 0.01% Tween-20, 100 mM NaCl, and 1 mM TCEP) with sonication. Approximately 1/3 of the insoluble protein was solubilized in pH 11 CAPS buffer. The remaining pellet was solubilized with 6 M GuHCl. The GuHCl-solubilized protein was diluted 10-fold in pH 11 CAPS buffer. Both the pH 11 soluble and GuHCl soluble fractions were concentrated to ∼20 mg/ml and purified further with a Superdex-200 column equilibrated with SEC buffer.

### Synthesis of bRNAs

The synthesis of branched RNAs proceeded on a 1 μmol scale using an ABI3400 DNA synthesizer and phosphoramidites and ancillary reagents obtained from Chemgenes Corporation. The synthetic protocol was followed exactly as per Method B of a previously published protocol with the modification that C, G, and U versions of monomer B were generated (39). Following synthesis and deprotection, the products were purified using a 24% polyacrylamide gel electrophoresis. A 40 ml gel was poured by mixing a gel stock solution with 300 μl 10% m/v ammonium persulfate and 30 μl TEMED and polymerized to a gel with a single long well. The crude samples (30 *As*) were applied to the wells in 40 μl dH_2_O and 60 μl deionized formamide after heating at 95 °C for 5 min. The gels were ran at 300 to 500 V over 2 to 3 h in 0.5× TBE buffer using a Hoefer SE600 Series Gel Apparatus. Following the run, the gels were examined using UV shadowing, and the main products were excised and soaked with water in a shaker (12–24 h). The water was filtered, lyophilized, and desalted using a 2.5 ml Sephadex G-25 column (Glen Research). The products were then quantified and verified for identity using an ESI-QTOF LCMS.

### Analytical ultracentrifugation

hDbr1, hDbr1-502, and hDbr1-399 were diluted to 0.9 *A_2_*_*80*_ in Q-A buffer and analyzed with a Beckman Coulter Optima ultracentrifuge at 40,000 RPMs. Data were analyzed with Ultrascan (40). Analysis was performed by the Center for Analytical Ultracentrifugation of Macromolecular Assemblies at the University of Texas Health Science Center at San Antonio.

### Elemental analysis of purified human Dbr1

Dbr1 samples were diluted in 2% nitric acid and analyzed with inductively coupled plasma mass-spectrometry at the University of Georgia Center for Applied Isotope Studies. To calculate molar equivalents, the μM concentration of metal ions was divided by the μM concentration of Dbr1 (determined with UV280 readings). Four independent samples of *E. coli* expressed hDbr1, one sample of insect expressed hDbr1, and one sample of metal-free apo-Dbr1 were analyzed.

### Nuclear magnetic resonance

*E. coli* and insect expressed hDbr1 were prepared at 50 μM in 10 mM phosphate buffer pH 7, with 5% D20. 1D 1H spectra with watergate water suppression were collected on a Bruker 7 MHz NMR instrument.

### Cell culture, transfection, and proteomic mass spectrometry

Wildtype 293 and two Dbr1-KO cell lines were cultured in DMEM with 10% FBS in 60 mM dishes. 800,000 cells were plated in triplicate in 60 mm dishes. The Dbr1 KO cells were generated using an Origene kit (KN200024) following the manufactures instructions. One hour after plating, each cell line was transfected with either wt-hDbr1 or RFP expression plasmids (2 μg plasmid/dish). Untransfected cells were maintained side by side, and each condition was tested in triplicate (3 cell lines, each with no transfection, Dbr1 transfection, or RFP transfection). After 48 h, cells were harvested with trypsin, washed twice with PBS, and frozen cell pellets were shipped to the Mass Spectrometry Core Laboratory at the University of Texas Health Science Center at San Antonio for lysis, protein quantification, and data-independent acquisition mass spectrometry. For Western blotting, a set of dishes was extracted with the Minute total protein extraction kit (Invent Biotechnologies).

Data were analyzed with Scaffold-DIA (Proteome Software). Two-fold changes were calculated relative to Dbr1-KO 1 cells.

### Western blotting

A custom rabbit Dbr1 antibody was used at 1:5k dilution (21). Rabbit TTDN1 antibody was from Novus (NBP2-31718), and GAPDH antibody was from Proteintech (10494-1-AP). Infrared goat anti-mouse (680 nm) and anti-rabbit (800 nm) secondary antibodies were from LiCor, and the blots were imaged with an Odyssey Clx scanner (LiCor).

### Sizing column analysis

Approximately 100 ug of Dbr1 was injected on a Superdex 200-Increase 10/300 column (Cytiva) equilibrated with SEC buffer. Drn1 was added at an equimolar ratio, and TTDN1 was added at a 2-fold molar excess.

### Metal-dependence assays

To generate metal-free apoenzyme, hDbr1 was exposed to high concentrations of metal chelators (10 mM EDTA and 10 mM NTA). After removing excess chelator with buffer exchange into assay buffer (=50 mM Hepes pH 7 100 mM NaCl 1 mM TCEP), the sample was verified to be metal free with ICP-MS and inactive in enzyme assays. Fe^2+^, Mn^2+^, or Zn^2+^ reconstitution experiments were performed under anaerobic conditions though the use of a fluorescent plate reader (Omega, BMG Labtech) housed in an anaerobic chamber (Coy labs). Similar experiments were performed as previously described in detail (15, 16). Apo Dbr1 was at a concentration of 1 μM, and the fluorescent bRNA substrate (33) was 25 μM. Metal ions were 0.5 to 100 μM. Steady-state rates (RFU/s) were calculated form the linear portion of progress curves and divided by the change in fluorescence from baseline to completed reactions to obtain μM/s rates, which were equivalent to turnover rates as the enzyme concentration was 1 μM.

### Fluorescence polarization assays

TTDN1 has a cystine residue at the C terminus; therefore, we used 5-iodoacetamido-fluorescein (Thermo 62246) to label TTDN1. The pH 11-soluble fraction of TTDN1 was brought to 10 mg/ml in SEC buffer, and 1/50th volume of 20 mg/ml 5-iodoacetamido-fluorescein in DMF was added. After 2 h at room temperature, the samples was passed over a PD10 desalting column (Cytiva) equilibrated with SEC buffer. The sample was further purified on a Superdex 200-Increase 10/300 column (Cytiva) equilibrated with SEC buffer. Purified, labeled TTDN1 was quantified by comparison to known standards on a Coomassie stained gel. Labeled TTDN1 (2.5 nM) was incubated with Dbr1 (3-fold dilution from 2.8 μM to 1 nM) or Drn1 (225 nM) in black 96-well half-area plates (Costar 3694). Proteins were diluted with SEC buffer. The fluorescence polarization was measured with a BioTek Synergy H1 plate reader equipped (Agilent). Plotting and curve fitting was performed with GraphPad (Prism).

### Branchpoint specificity assays

The bRNAs were diluted to 5 μM in assay buffer (50 mM Hepes pH 7, 100 mM NaCl, 1 mM TCEP). Dbr1 was diluted to 10 μM. Each reaction consisted of 25 μl of bRNA. Prior to enzyme addition, a 3 μl aliquot was removed to serve as the t = 0 point. 1/10 volume 10 μM Dbr1 was added (1 μM final), and 3 μl aliquots were removed at the indicated points (Fig. 5*B*). Timepoints were frozen at −80 until the last point was collected, at which point 12 μl of water were added, and the samples heated at 75 °C for 2 min. Samples were then frozen prior to analysis on a Bioanalyzer instrument using the Small RNA kit (Agilent). To quantify the disappearance of full-length bRNA, the peak heights were determined using the instrument software (Expert 2100, Agilent). The t = 0 points were normalized to 5 μM, and the curves were plotted with GraphPad (Prism). The rates were estimated using the SLOPE function of Excel. The normalized rates were then plotted with GraphPad (Fig. 5*E*).

### Steady state kinetics of Dbr1 with TTDN1 and Drn1

Fluorescent bRNA substrate was prepared in assay buffer at concentrations from 20 nM to 10 μM. Dbr1 was diluted to 1 μM in enzyme buffer (50 mM Hepes pH7, 100 mM NaCl, 1 mM TCEP, 0.02 mg/ml BSA, 0.01% Tween-20). Drn1 and TTDN1 (Gu-HCl solubilized) was included at 10 μM. These mixtures were then diluted 100-fold in enzyme buffer, to a final concentration of 10 nM hDbr1, 100 nM Drb1, and 100 nM TTDN1. The diluted samples and substrate dilutions were mixed 20 μl + 20 μl, in triplicate, in black 96-well half-area plates (Costar 3694). Fluorescence (488 nM) was measured every 20 s for 5 h using the 'sweep' mode on a BioTek Synergy H1 plate reader. Initial rates were calculated using Gensys3 software (BioTek). Curves were fit with an allosteric sigma equation to estimate K_m_, k_cat_, and the Hill coefficient (n_H_), or a modified Michalis-Menten equation to estimate the K_i_ of substrate inhibition (41, 42).

### Kinetic analysis of Dbr1 truncations

Protein expression was as described above (Bacterial expression), but the culture volumes were 10 mls, and the lysates were prepared with BugBuster. The lysates were then diluted 1:100 and mixed with fluorescent bRNA substrate (0.2 μM final concentration). The concentration of Dbr1 in the lysates was unknown, so the steady state debranching rates were normalized to full-length Dbr1. The results from three independent experiments are plotted in Figure 2*B*.

## Data availability

The raw data generated during all experiments are available upon request from the author.

## Supporting information

This article contains supporting information.

## Conflict of interest

The authors declare no conflict of interest with the contents of this article.

## Supporting information

Supporting Figure 1 *A*, 1D proton NMR shows that TTDN1 contains mostly random-coil polypeptide at pH 4.5 (*blue*), but increased structure in the 8.2 ppm region at pH 7.5 (*red*). *B*, circular dichroism analysis of Dbr1 (*red*) and TTDN1 (*blue*) at pH 7.4. Consistent with the 1D-NMR, a minima at 220 nm suggests that TTDN1 has some helical character. *C*, an alpha-fold model of TTDN1 also predicts some helical content in a mostly disordered protein. Colored by pLDDT score, with *blue* = high confidence and *red* = low confidence. NMR, nuclear magnetic resonance.
