# Supplementary figures and images for: Activation of human RNA lariat debranching enzyme Dbr1 by binding protein TTDN1 occurs though an intrinsically disordered C-terminal domain
Source: J Biol Chem. 2023 Jul 26;299(9):105100. doi: 10.1016/j.jbc.2023.105100 (PMC10470207; doi:10.1016/j.jbc.2023.105100)

Supporting Figure1

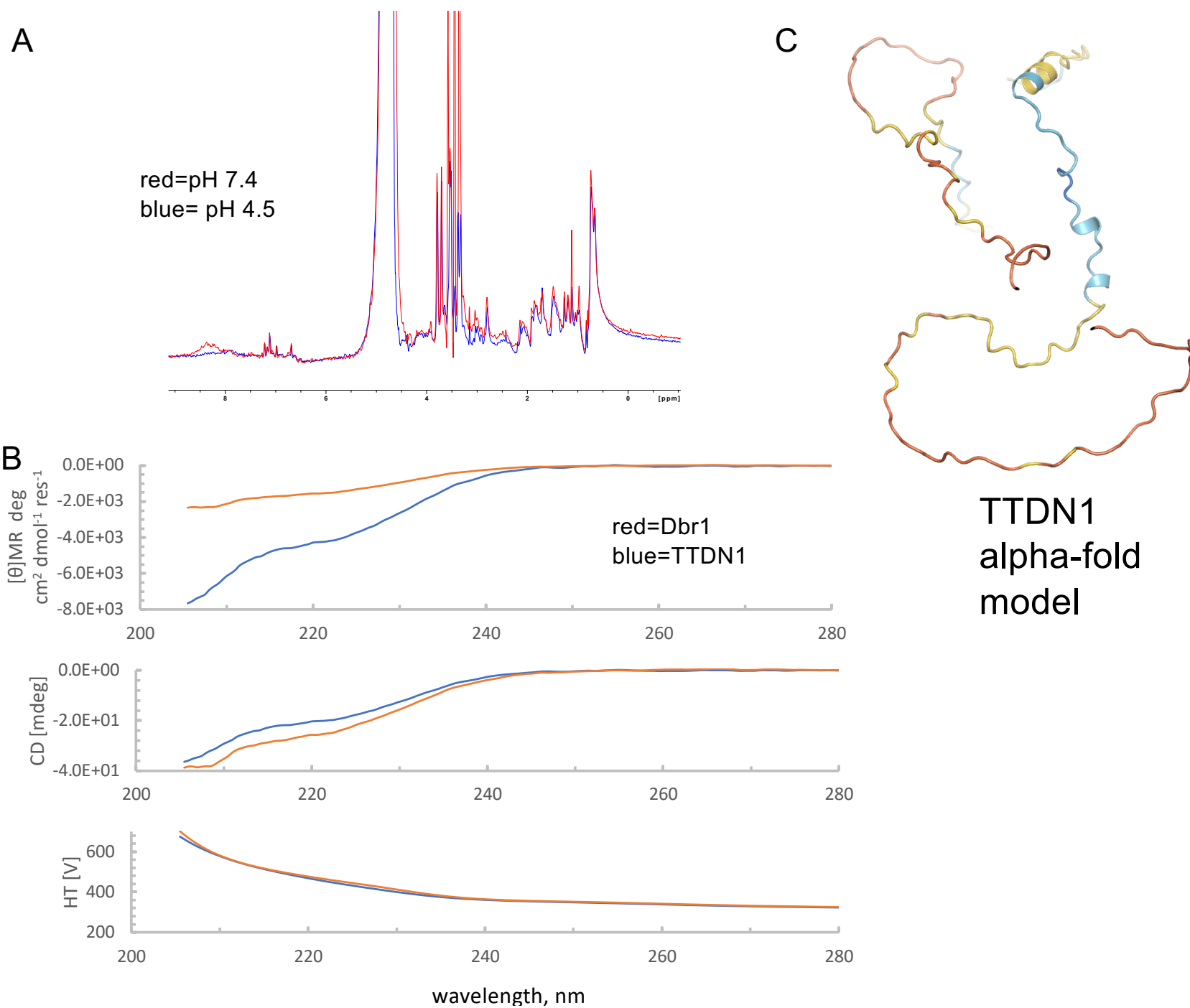

Supplement: Supporting Figure 1 — A, 1D proton NMR shows that TTDN1 contains mostly random-coil polypeptide at pH 4.5 (blue), but increased structure in the 8.2 ppm region at pH 7.5 (red). B, circular dichroism analysis of Dbr1 (red) and TTDN1 (blue) at pH 7.4. Consistent with the 1D-NMR, a minima at 220 nm suggests that TTDN1 has some helical character. C, an alpha-fold model of TTDN1 also predicts some helical content in a mostly disordered protein. Colored by pLDDT score, with blue = high confidence and red = low confidence. NMR, nuclear magnetic resonance. [file mmc1.pdf]
